# Dermal γδ T-Cells Can Be Activated by Mitochondrial Damage-Associated Molecular Patterns

**DOI:** 10.1371/journal.pone.0158993

**Published:** 2016-07-12

**Authors:** Martin G. Schwacha, Meenakshi Rani, Susannah E. Nicholson, Aaron M. Lewis, Travis L. Holloway, Salvador Sordo, Andrew P. Cap

**Affiliations:** 1 Department of Surgery, The University of Texas Health Science Center at San Antonio, Texas, United States of America; 2 Blood Research, United States Army Institute of Surgical Research, Fort Sam Houston, Texas, United States of America; Georgia Regents University, UNITED STATES

## Abstract

**Background:**

Gamma delta T-cells have been shown to be important to the early immunoinflammatory response to injury, independent of infection. This unique T-cell population acts to regulate cell trafficking and the release of cytokines and growth factors. We propose this sterile inflammatory response is in part associated with damage associated molecular patterns (DAMPs) generated by major injury, such as burn, and mediated via toll-like receptors (TLRs). It is unknown whether DAMPs can activate resident γδ T-cells that reside in skin.

**Methods:**

Gamma delta T-cells were isolated from the skin of male C57BL/6 mice by enzymatic digestion. Mitochondrial DAMPs (MTDs) were generated from mitochondria isolated from mouse livers by sonication and centrifugation. Dermal γδ T-cells were incubated with MTDs (0–500 μg/ml) for 24 hr and cells and supernatants were collected for analysis.

**Results:**

MTDs activated dermal γδ T-cells, as evidenced by increased TLR2 and TLR4 expression following in vitro exposure. MTDs also induced the production of inflammatory cytokines (IL-1β, IL-6), and growth factors (PDGF and VEGF) by γδ T-cells.

**Conclusions:**

These findings herein support the concept that MTDs released after tissue/cellular injury are capable of activating dermal γδ T-cells. We propose that the activation of this unique T-cell population is central in the initiation of sterile inflammation and also contributes to the subsequent healing processes.

## Introduction

T-cells of the γδ T-cell receptor (TCR) lineage are involved in a wide variety of disease processes [1–3]. This unique T-cell subset has been shown to have a critical role in inflammation and tissue repair [4–7]. Work from our laboratory supports the concept that γδ T-cells play a central role in the early inflammatory and immune response to burn injury at multiple levels including wound healing and end organ injury [8–11]. The mechanism(s) and mediator(s) responsible for mobilization and activation of this T-cell subset are under such conditions are unclear.

The Danger Theory as proposed by Matzinger [12,13] provides a key link between tissue injury and the innate immune system. The theory suggests that one of the functions of the innate immune system is to prevent and recognize attack from harm. In this regard, the mechanism of cell death determines whether an immune response is initiated. Controlled cell death, or apoptosis, does not lead to the generation of damage associated molecular patterns (DAMPs); however, cell death by necrosis (often associated with tissue injury) generates DAMPs, which in turn activate the innate immune system. This activation occurs via pattern recognition receptors (PPRs). Interestingly pathogen-associated molecular patterns (PAMPs) and DAMPs have similar conserved hydrophobic portions that engage the same PRRs to elicit comparable responses [14]. Potent immune activation can be mediated via PRRs, such as toll-like receptors (TLRs), which represent a key link between tissue injury, infection, and inflammation. Potential DAMPs involved in the activation of the immune system via TLRs include mitochondrial DNA, HMGB-1 and S100A8 [15–17]. The role of TLRs in tissue injury, infection, and inflammation provides an important link between these processes. Hauser and co-workers [18] demonstrated that mitochondrial DAMPs, released by cellular disruption after trauma, are present in the circulation and activate neutrophils. Damage of cells, such as occurs with trauma, can induce cell death and necrosis. Necrotic cells can spill their intracellular components, including mitochondria-related molecules (i.e., mitochondrial DAMPs) which are involved in initiating inflammatory responses. These mitochondrial DAMPs include mitochondrial DNA, N-formyl peptides, cardiolipin, cytochrome C, carbamoyl-phosphate synthase 1 and ATP which are recognized by a number of different receptor types, including TLRs [18–21].

Recently we have demonstrated that splenic γδ T-cells can be also activated by DAMPs leading to increased TLR expression and cytokine, chemokine and growth factor release [22]. While these finding support the concept of DAMP-mediated activation of γδ T-cells after injury, unique subsets of γδ T-cells exist in the skin (as opposed to traditional lymphoid organs such as the spleen), which may show different patterns of activation [23,24]. Our previous work in a preclinical model of burn injury has demonstrated that dermal γδ T-cells are involved processes essential in the healing of the burn injury site including cytokine, chemokine and growth factor production and cellular recruitment, [7,10,25–27] The mechanism by which these γδ cells are activated is unclear. The current study was undertaken to determine whether dermal γδ T-cells are capable of being activated for these functions by mitochondrial DAMPs and thus provide a potential mechanism by which γδ T-cell are activated after tissue injury, such as with burn.

## Materials and Methods

### Animals

C57BL/6 male mice (12–14 week old; Jackson Laboratories, Bar Harbor, ME, USA) were used for all experiments. Prior to experimentation all mice were acclimatized for at least one week. The protocol were approved by the University of Texas Health Science Center at San Antonio Institutional Animal Care and Use Committee (IACUC) and conducted in compliance with the Animal Welfare Act, the implementing Animal Welfare Regulations, and the principles of the Guide for the Care and Use of Laboratory Animals. The mice were euthanized by overdose of isoflurane anesthetic and subsequent bilateral thoracotomy.

### Isolation of Dermal γδ T-Cells

Dermal γδ T-cells used for these studies were isolated from the skin of male C57Bl/6J mice, as described in detail previously [27]. Briefly, depilated skin samples were collected to the level of the musculofascia by sharp dissection, which included the submucosal layer. The skin samples were washed and minced into small pieces (2–3 mm) in sterile PBS containing 50 U/mL each of penicillin and streptomycin (Gibco ThermoFisher Scientific, Waltham, MA USA). The minced skin was digested overnight in dispase II (0.05%, Roche Diagnostics Corp., Indianapolis, IN USA). Following the overnight incubation the skin samples were minced further into smaller pieces and subjected to digestion by agitating in trypsin-GNK (0.3%, Glucose/dextrose, NaCl and KCl buffer, Sigma-Aldrich, St. Louis, MO USA) for 30 min at 37°C in a water bath shaker. The digestion reaction was stopped by the addition of heat-inactivated fetal bovine serum (FBS, Gibco ThermoFisher Scientific). The dissociated cells were sieved through a 100 μm mesh and resuspended in RPMI containing 10% heat-inactivated FBS, 50 μM of 2-Mercaptoethanol (Sigma-Aldrich), 2 mM of L-glutamine (Gibco ThermoFisher Scientific), 1 mM of sodium pyruvate (Gibco ThermoFisher Scientific), 100 μM Non-essential amino acids (Gibco ThermoFisher Scientific), 50 U/mL penicillin and 50 μg/mL streptomycin supplemented with 10 U/mL murine recombinant IL-2 (BD Biosciences, San Jose, CA USA). Dermal T-cells were isolated from the dermal cell suspension using an EasySep^™^ Mouse T-cell enrichment kit according to the manufacturer’s recommendations (Stemcell Technologies, Vancouver, BC, Canada). The enriched T-cell population was greater than 95% CD3^+^and cell viability was routinely greater than 80% as determined by trypan blue exclusion.

### Generation of Mitochondrial Damage-Associated Molecular Patterns (MTDs)

MTDs were generated as described in our previous publication in accordance with the methods originally published by Zhang et al. [18,22]. Briefly, mitochondria were isolated from mouse livers using a mitochondria isolation kit (Thermo Scientific, West Palm Beach, FL, USA), according to the manufacturer’s recommendations. The mitochondria were disrupted using five freeze/thaw cycles in liquid nitrogen. The resulting disrupted mitochondrial suspensions were centrifuged at 12,000g (10 min at 4°C) and then at 100,000g (30 min at 4°C). The residual supernatants which contained the MTDs were used for the experiments. The BCA Protein Assay (Thermo Scientific) was used to determine the protein concentration.

### In Vitro Stimulation of Dermal γδ T-Cells

T-cells (1x10^6^/mL) were cultured in a 12-well plate in complete RPMI (RPMI 1640 containing 10% heat-inactivated FBS, 5 μg/mL gentamycin and 100 mg/mL of streptomycin and penicillin; GibcoBRL, Grand Island, NY, USA) for 24 hr with MTDs (0–500 μg/mL), as described in detail previously [22]. The concentrations of MTDs used in the study herein are based on this previous work with splenic γδ T-cells. Following the 24 hr incubation the cells were collected for phenotypic analysis by flow cytometry. Cell-free supernatants were collected from the culture plates and stored at -80°C for later analysis of cytokine and growth factor content.

### Cell Phenotyping by Flow Cytometry

T-cells were phenotyped for γδ T-cell receptor (TCR) expression and their TLR expression using standard flow cytometry techniques. The γδ T-cells and MTDs cultured cells were washed in staining buffer (PBS with 0.2% BSA and 0.09% NaN_3_) and treated with Fc-blocking antibody (anti-CD16/CD32, BD Biosciences) for 15 min. The cells were subsequently stained with conjugated antibodies as follows: anti-CD3 (APC) in combination with anti-δ TCR (FITC), anti-TLR2 (PE) and anti-TLR4 (PE-Cy7). After 30 min of incubation on ice, the cells were washed and resuspended in staining buffer. Appropriate isotype antibody controls were used. The data were acquired using a LSRII flow cytometer (BD Biosciences) and FlowJo software (Tree Star, Ashland, OR, USA) was used for analysis. A minimum of 50,000 events was collected and gating was on live cells according to forward- and side-scatter properties.

### Cytokines and Growth Factors Analysis

Cytokines and growth factors levels in the cell culture supernatants were assayed using the Bio-Plex (BioRad, Hercules, CA, USA) system in accordance with the manufacturer’s recommendations. The following factors were assessed: IL-1β, IL-6, IL-10, fibroblast growth factor (FGF), platelet-derived growth factor (PDGF) and vascular endothelial growth factor (VEGF).

### Statistical Analysis

Data are expressed as mean ± SEM (n = 5mice/group). The cell isolation and cultures were conducted in 2 independent experiments on separate days. The data were analyzed by One-way repeated measures of the variance with Bonferroni’s t-test for multiple comparisons versus a control group using Sigma Plot 11.0 (Jandel Scientific, San Rafael California, USA). A *p*-value of ≤ 0.05 was considered to be statistically significant for all analyses.

## Results

### MTDs Increase TLR expression by Dermal γδ T-Cells

**Fig 1:**
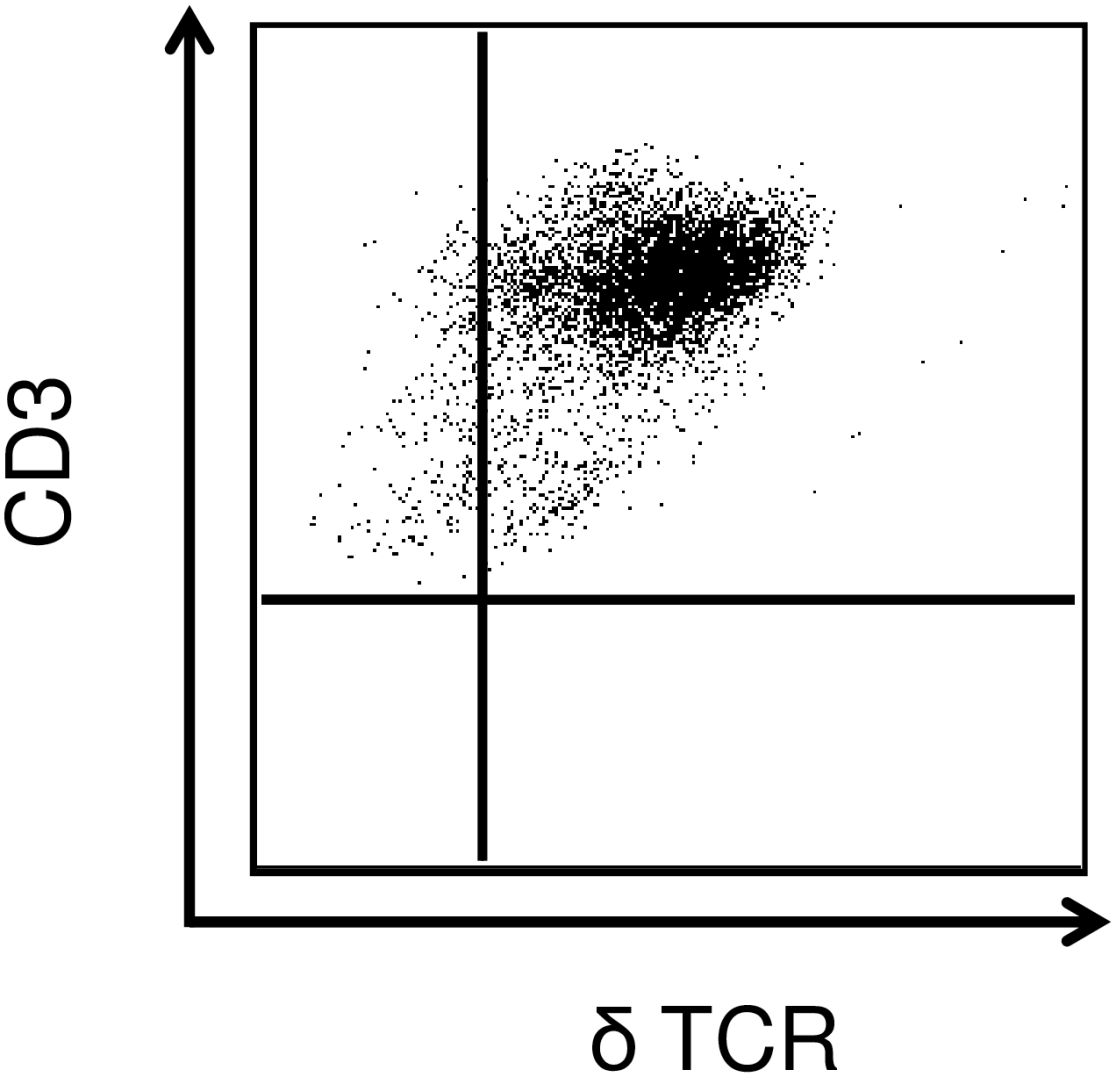
Characterization of dermal T-cell*s*. Dermal T-cells were isolated as described in the materials and methods and phenotyped for CD3 and δTCR expression by flow cytometry. A representative dot plot for CD3 and δTCR expression of the CD3^+^ population is shown.

**Fig 2:**
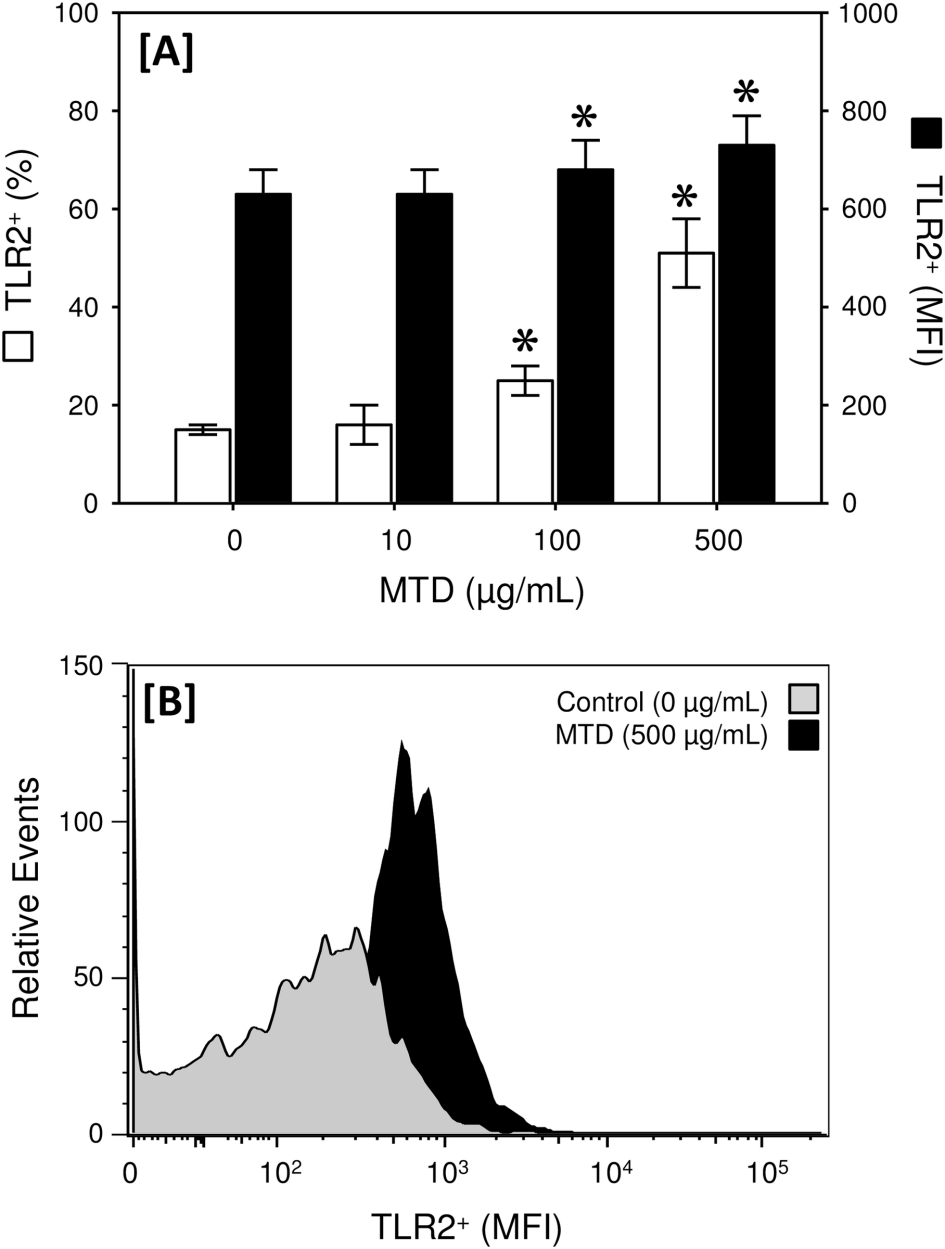
MTDs increase TLR2 expression by dermal γδ T-cell*s*. Skin T-cells were incubated with MTDs (0–500 μg/mL) and TLR2 expression on the γδ T-cell population was determined by flow cytometry as described in the materials and methods. Panel A shows the percentage of the cells positive for TLR2 expression (TLR2^+^) □ and the mean fluorescence intensity (MFI) of the TLR2^+^ cells ■. The data are Mean ± SEM for cells from 5 mice. Panel B shows a representative histogram plot for TLR2 expression by control cells and following stimulation with 500 μg/mL MTDs. *p<0.05 as compared with control (0 μg/mL).

The dermal T-cell preparation was 96% CD3^+^ δ TCR^+^ (Fig 1). Approximately 15% of the γδ T-cells were positive for TLR2 expression in the absence of stimulation (Fig 2A). MTD stimulation dose-dependently increased the percentage of cells expressing TLR2. Approximately 50% of the γδ T-cells expressed TLR2 on their surface after stimulation with 500 μg/mL MTDs. Similarly, MTD stimulation elevated the mean fluorescence intensity (MFI) for TLR2 expression at concentrations of 100 and 500 μg/mL. The uniformity of TLR2 expression was also increased by MTDs as evidenced by a decreased MFI range (Fig 2B).

**Fig 3:**
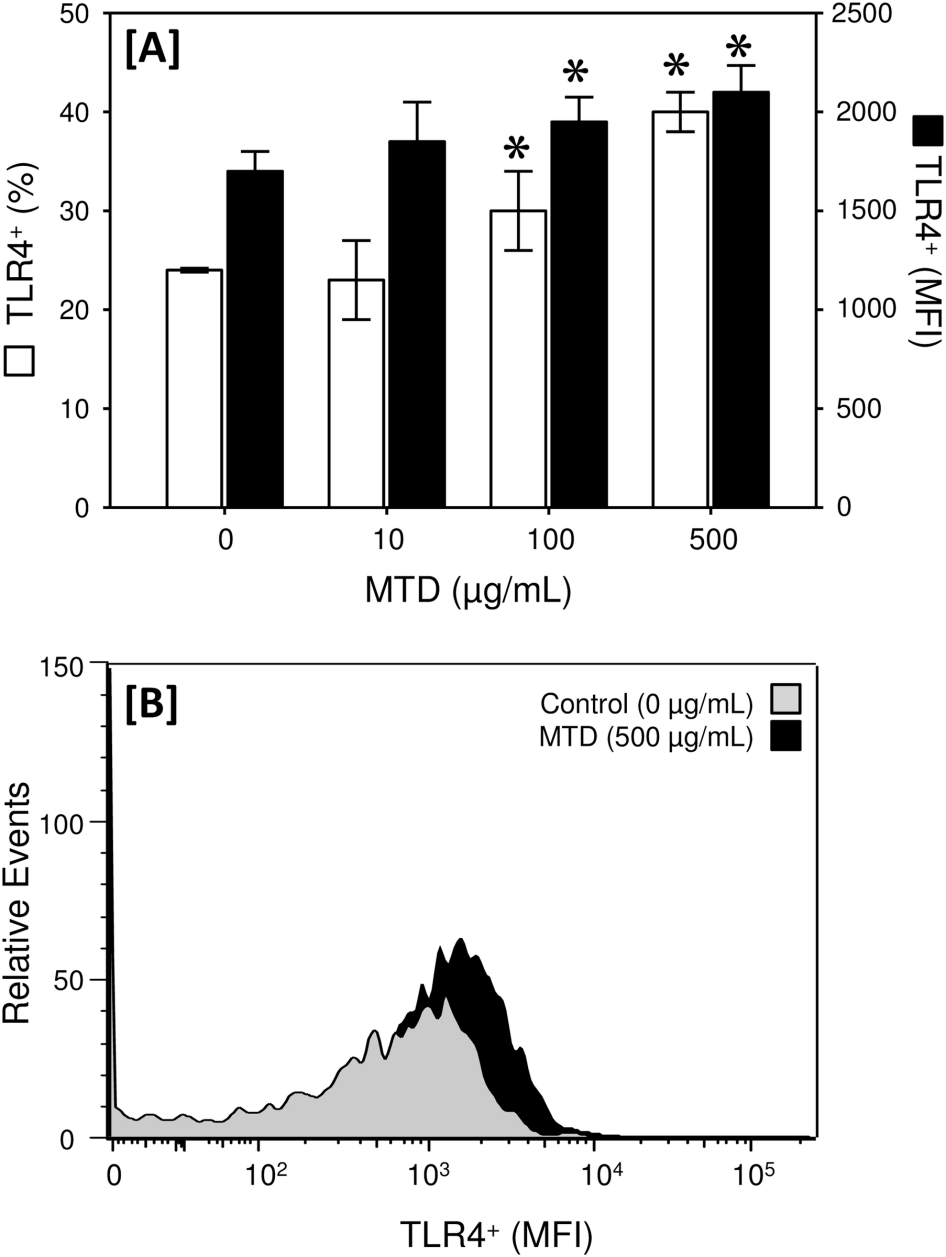
MTDs increase TLR4 expression by dermal γδ T-cells. Skin T-cells were incubated with MTDs (0–500 μg/mL) and TLR4 expression on the γδ T-cell population was determined by flow cytometry as described in the materials and methods. Panel A shows the percentage of the cells positive for TLR4 expression (TLR4^+^) □ and the mean fluorescence intensity (MFI) of the TLR4^+^ cells) ■. The data are Mean ± SEM for cells from 5 mice. Panel B shows a representative histogram plot for TLR4 expression by control cells and following stimulation with 500 μg/mL MTDs. *p<0.05 as compared with control (0 μg/mL).

Approximately 24% of the γδ T-cells were positive for TLR4 expression in the absence of stimulation, which was significantly greater (p<0.05) than that for TLR2 (14.9 ± 0.5% vs. 24.4 ± 0.2% for TLR2 and TLR4, respectively; mean ± SEM, n = 5/group) (Fig 3A). Similar to TLR2, MTD stimulation dose-dependently increased the percentage of cells expressing TLR4; however, the increase was not as profound. Approximately 40% of the γδ T-cells expressed TLR2 on their surface after stimulation with 500 μg/mL MTDs. MTD stimulation also elevated the MFI for TLR4 expression at concentrations of 100 and 500 μg/mL, but the uniformity of TLR4 expression (MFI range) was unaltered by MTDs. The uniformity of TLR2 expression was also increased by MTDs as evidenced by a decreased MFI range (Fig 3B).

**Fig 4:**
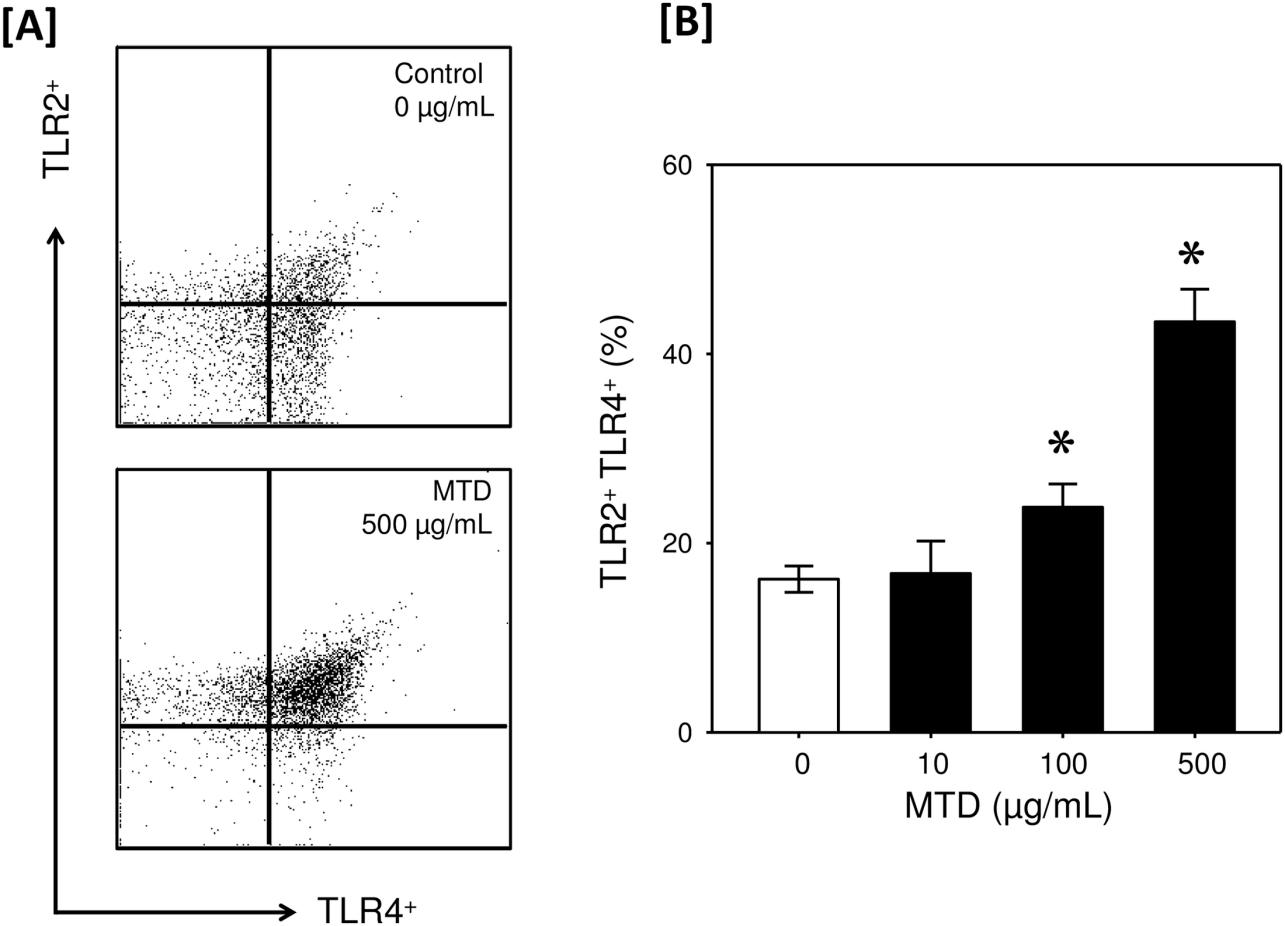
MTDs increase co-expression of TLR2 and TLR4 by dermal γδ T-cells. Skin T-cells were incubated with MTDs (0–500 μg/mL) and TLR2 and TLR4 co-expression on the γδ T-cell population was determined by flow cytometry as described in the materials and methods. Panel A shows a representative scatter plot for TLR2 and TLR4 expression on dermal γδ T-cells under control conditions and following stimulation with 500 μg/mL MTDs. Panel B shows the percentage of γδ T-cells positive for both TLR2 and TLR4. Data are Mean ± SEM for cells from 5 mice. *p<0.05 as compared with control (0 μg/mL).

Further analysis of TLR expression on the γδ T-cells revealed that under control or unstimulated conditions only 16% of the cells were positive for both TLR2 and TLR4 expression with a marked proportion of the cells only positive for one of the TLRs (Fig 4A). Following stimulation with MTDs a profound increase in uniformity in TLR expression was observed and the percentage of double positive cells significantly increased by 50% with 100 μg/mL MTD stimulation and by 100% with 500 μg/mL MTD stimulation (Fig 4B).

### MTDs Stimulate Dermal γδ T-Cells to Produce Selected Cytokines and Growth Factors

**Fig 5:**
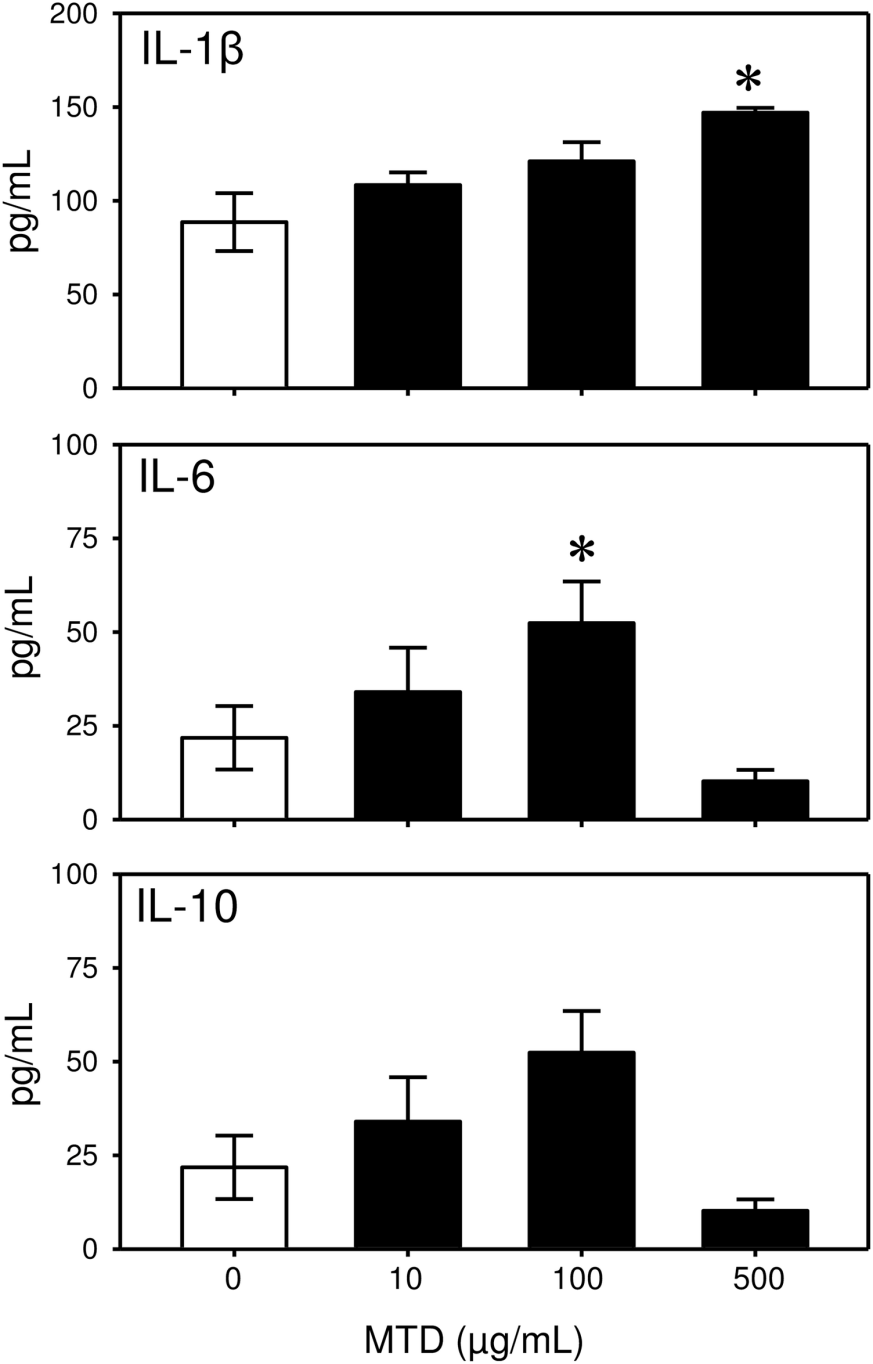
MTDs increase cytokine release by γδ T-cells. Skin T-cells were incubated with MTDs (0–500 μg/mL) and cytokine (IL-1β, IL-6, IL-10) levels were determined by Bio-Plex assay as described in the materials and methods. Data are presented as median with the 25th and 75^th^ percentile for cells from 5 mice. * p<0.05 as compared with control (0 μg/mL MTD).

Cytokine and growth factor levels were somewhat variable between the cultures (Table 1). MTD stimulation of the dermal γδ T-cells induced a significant increase in the production of the pro-inflammatory cytokines IL-1β and IL-6, but not the anti-inflammatory cytokine IL-10 (Fig 5). IL-1β release was increased approximately 50% by MTDs at a concentration of 500 μg/mL. In contrast, IL-6 release was elevated 5-fold at a lower concentration of MTD (i.e., 100 μg/mL). High concentrations of MTD (500 mg/mL) were ineffective in stimulating IL-6 release by the cells, as levels were comparable to that observed in unstimulated cultures. While a trend towards elevated release of IL-10 was observed with MTD stimulation, it was not statistically significant.

**Table 1:** Cytokine and Growth Factor Levels in Cell Culture Supernatants^a^.

	Unstimulated (0 μg/mL)			Maximal MTD response^b^		
	Mean	Median	Range	Mean	Median	Range
IL-1β	89	98	29–117	147	143	143–155
IL-6	22	15	3–52	53	43	30–80
IL-10	18	19	6–21	30	35	19–38
FGF	62	69	22–94	61	75	23–88
PDGF	1,022	671	417–1,918	4,164	3,137	1,504–6,893
VEGF	205	193	82–356	606	607	484–715

^a^Data are the mean, median and range of cytokine and growth factor levels (pg/mL) for dermal T-cell cultures as described in the materials and methods (n = 5/group).

^b^The values shown are for IL-6, IL-10 and PDGF are for 100 μg/mL MTD and the values shown for IL-1β, FGF and VEGF are for 500 μg/mL MTD.

**Fig 6:**
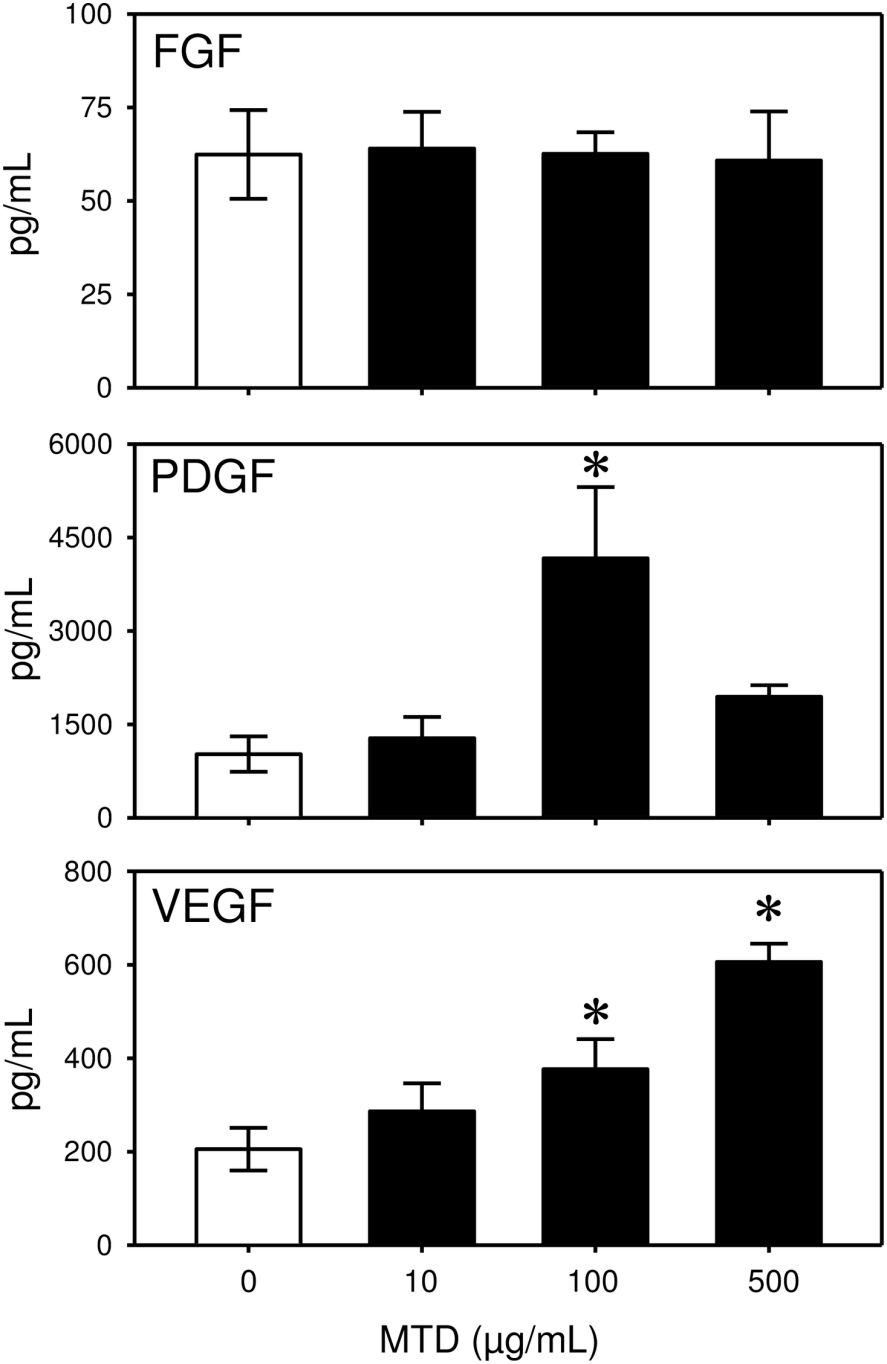
MTDs increase growth factor release by γδ T-cells. Skin T-cells were incubated with MTDs (0–500 μg/mL) and cytokine (FGF, PDGF, VEGF) levels were determined by Bio-plex assay as described in the materials and methods. Data are presented as median with the 25th and 75^th^ percentile for cells from 5 mice. * p<0.05 as compared with control (0 μg/mL MTD).

The release of selected growth factors after MTD stimulation was also observed (Fig 6). MTDs did not stimulate an increase in the release of FGF by γδ T-cells at any concentrations tested. In contrast, the release of both PDGF and VEGF were increased by MTDs. In the absence of MTD stimulation, significant levels of PDGF were present (Table 1). Stimulation of the cells with MTDs at 100 μg/mL resulted in a 6-fold increase in PDGF release. VEGF release by the cells was increased dose-dependently. VEGF release was increased 2-fold and 4-fold by 100 and 500 μg/mL MTDs.

## Discussion

Activation of the innate immune system is an early step in the response to infection or injury and is essential in the clearance of the infection, injured tissue and healing. Macrophage and neutrophils are considered the primary effector cells of the innate immune system; however, a unique T-cell population, γδ T-cells, are also important in innate immunity and play an important role in inflammation and tissue repair [4,5,7,28]. Previous studies have indicated that these T-cells are activated after injury [7,29,30]. Our findings presented herein support the concept that dermal γδ T-cells can be activated by mitochondrial DAMPs (which are released after tissue injury), thus providing a potential mechanism by which this unique T-cell population is activated under such conditions.

Cellular injury can lead to release of intracellular molecules referred to as DAMPs [31,32]. These mediators can be derived from a large array of cellular components which includes the cytosol, endoplasmic reticulum, nucleus, plasma membrane, and mitochondria [33]. The existence of common recognition patterns for DAMPs and PAMPs on TLRs supports that mitochondria can serve as an important source of DAMPs after injury. A wide range of molecules of mitochondrial origin have been identified as DAMPs which are recognized by a number of different receptor types, including TLRs [18,20,21]. Under homeostatic conditions, apoptosis does not lead to DAMP generation; however, cell death by necrosis, which is often associated with tissue injury, leads to DAMP generation. These DAMPs in part via interaction with immune cell TLRs lead to activation of the innate immune system. TLRs provide a link between tissue injury, infection, and inflammation.

The majority of T-cells found in the epidermis of mice are of the γδ TCR lineage [34,35]. TLR expression by γδ T-cells has been established [36] and recent findings have shown that the expression of TLR2, TLR4 and TLR9 by dermal γδ T-cells is increased after burn injury [27]. Since, DAMPs interact via TLRs they are likely to represent an important mechanism by which γδ T-cells are activated in the injured skin and initiate sterile inflammation. The inflammatory process is considered to be instrumental in supplying growth factors, cytokines, and other factors essential to wound repair [37,38]. It is believed that dermal γδ T-cells survey for general signs of injury/stress rather than recognition of specific antigens. In this regard, γδ T-cells are capable of recognizing antigens expressed by injured keratinocytes and subsequently produce chemokines and growth factors [24,39]. Jameson et al. [28] have shown defects in keratinocyte growth and tissue reepithelialization in mice lacking in γδ T-cells. Similarly, our laboratory has demonstrated that such mice have delayed healing of the burn wound site that was associated with suppressed growth factor levels and cellular infiltration [10,11]. We have begun to analyze the dermal γδ T-cell population in our preclinical mouse burn model. These studies have characterized these cells as follows: They are predominately CD4^-^CD8^-^ and shift to a CD4^+^ population in the wound [40]; they express TLR2, 4, and 9, which is upregulated in the wound site [27]; they express increased levels of the activation marker CD69 in the burn wound [27,40]; they promote a Th2 and Th17 cytokine profile in the wound with elevated levels of IL-10 and IL-17 respectively [41]; and they are essential in the recruitment of both T-cells and myeloid cells to the burn wound [27,42].

Studies have shown that endogenous TLR ligands or DAMPs only activate genes involved in inflammation and tissue repair, whereas pathogenic TLR ligands (PAMPs) also activate acquired immunity [43]. Activation of γδ T-cells after injury (independent of infection) has been shown to be essential in efficient wound healing [7,28,44]. Thus, DAMP-mediated activation of TLR signaling by dermal γδ T-cells is likely to be essential in their central role in regulating wound inflammation and healing. Our previous findings [22] have shown that splenic γδ T-cells are activated by MTDs; however, variations in γδ TCRs exist between lymphoid organs and the skin [23,24], thus warranting the investigation of the responsiveness of dermal γδ T-cells to DAMPs. Our findings herein confirm that dermal γδ cells can be activated by MTDs; however, the response differed to some degree from that of splenic γδ T-cells previously observed.

We observed a degree of specificity with regard to the MTD-mediated cytokine release. In particular, IL-1β and IL-6 were up-regulated, whereas IL-10 was not significantly affected. Likewise growth factor release was elevated for PDGF and VEGF, but not FGF. These response differ from that observed with MTD-mediated activation of splenic γδ T-cells where elevations in IL-10 and FGF were observed [22]. This points to the importance of the heterogeneity and tissue specificity of γδ T-cells [23,24]. We observed that basal levels of FGF were already quite high (~5000 pg/mL) in the absence of MTDs, and it can be speculated that further induction was not possible. In addition, IL-10 likely acts to quell the initial pro-inflammatory response (IL-1β, IL-6) and since the cells were only maintained in culture for 24 hrs sufficient time was not allowed to generate a statistically significant response to MTDs and/or the pro-inflammatory cytokines generated by MTDs. In addition, the decrease in IL-6 levels at the highest concentration of MTDs may be related to the anti-inflammatory properties of IL-10. Nonetheless issues of toxicity cannot be ruled out, as the cells were not assessed for apoptosis, necrosis or other markers of cell death after culture.

The association between elevated TLR2 and TLR4 expression of increased cytokine and growth release after MTD stimulation is only associative and the role of other receptors cannot be ruled out. Others have also shown a similar associative relationship between T-cell function and TLR expression in a murine burn model [45]. In contrast, others have shown in a clinical study of vasculitis that elevated TLR expression did not affect *ex vivo* cytokine production by NK cells [46]. Additional experimental studies are needed to address these issues, including *in vivo* models of MTD-induced inflammation. The current study herein is limited by the analysis of only TLR2 and TLR4, consistent with our previous study with MTDs [22]. While we have previously shown that dermal γδ T-cells express TLR9 [27], which is the receptor for mitochondrial DNA, other TLRs, such as TLR3 have not been evaluated. The effects of MTDs on the expression of these other TLRs would be of interest and their evaluation in future studies is warranted.

## Conclusions

Our findings presented herein demonstrate that dermal γδ T-cells express TLRs and can be activated by endogenous ligands generated by tissue injury (i.e., mitochondrial-derived DAMPs). These DAMPs are likely to be at high concentrations at a site of tissue injury. The activation of this unique T-cell population can lead to the release of specific cytokines and growth factors capable of regulating the early stages of tissue remodeling. The observed upregulation of TLRs on dermal γδ T-cells after exposure to MTDs likely makes them more responsive to subsequent activation via TLR-dependent pathways. We hypothesize that tissue injury-induced release of MTDs is an important mechanism for the initiation of γδ T-cell dependent sterile inflammation and subsequent tissue remodeling responses.
